# RORα controls inflammatory state of human macrophages

**DOI:** 10.1371/journal.pone.0207374

**Published:** 2018-11-28

**Authors:** Neda Nejati Moharrami, Erlend Bjørkøy Tande, Liv Ryan, Terje Espevik, Victor Boyartchuk

**Affiliations:** 1 Centre of Molecular Inflammation Research (CEMIR), Department of Clinical Research and Molecular Medicine (IKOM), Faculty of Medicine and Health Sciences (MH), Norwegian University of Science and Technology (NTNU), Trondheim, Norway; 2 Clinic of Cardiology, St. Olavs Hospital HF, Trondheim University Hospital, Trondheim, Norway; Tufts University School of Medicine, UNITED STATES

## Abstract

ROR family of nuclear receptor transcription factors forms nodes connecting metabolic and inflammatory signaling pathways. The RORα members of the family have intrinsic transcriptional activity and they are involved in both activation and repression of a wide range of genes. The role of RORα in control of inflammation has been extensively studied using animal models but its function in human cells is not as well understood. To address this shortcoming, we analyzed how RORα is shaping the inflammatory state of human macrophages. Using CRISPR-Cas9 system, we deleted *RORA* in THP-1 human monocytic cell line. In mutant cells we observed a dramatic increase in basal expression of a subset of NF-κB regulated genes, including TNF, IL-1β and IL-6, at both transcriptional and translational levels. Furthermore, *RORA*-deletion cells produced notable amounts of pro-IL-1β even in the absence of LPS stimulation. Subsequent LPS stimulation induced cleavage of pro-IL-1β to mature form. Our RNAseq analysis further confirmed the key role of *RORA* in setting the inflammatory state of macrophages and defined the set of differentially regulated genes. Overall, our data provides evidence supporting the anti-inflammatory function of RORα in human macrophages.

## Introduction

RAR-related Orphan Receptors (RORs) are members of nuclear receptor (NR) transcription factor family that tie together metabolic and inflammatory signaling pathways. In mammals, there are three major isotypes of RORs: α, β, and γ. All three of these isotypes are further diversified by alternative splicing and different promoter usage producing numerous cell-type specific isoforms differing at their N-terminus [1–4]. Recent studies suggested that oxysterols, cholesterol, 7-dehydroxycholeterol, hydroxyderivatives of vitamin D and lumisterol derivatives can act as RORα ligands while all-trans retinoic acid have been shown to stimulate RORβ activity [5–10]. Because of this, ROR NRs have now been deorphanized. Notably, simvastatin, a cholesterol synthesis inhibitor, has been shown to decrease viability of macrophages and this process can be reversed by SR1001, a synthetic ligand of RORα [11].

Human macrophages produce mostly RORα [12] encoded by the *RORA* gene. RORα, also known as NR1F1, is also widely expressed in other tissues, including brain, thymus, heart, vessels, liver, etc. It is a multifunctional transcription factor that has been shown to play important roles in cerebellar development, osteogenesis and atherogenesis [13–19]. RORα has four isoforms, RORα1–4, that share DNA-binding domain (DBD) and ligand-binding domain (LBD). These isoforms demonstrate different tissue distribution. While RORα1 and RORα4 are expressed ubiquitously [20, 21], RORα3 expression in humans appears to be restricted to testis and RORα2 isoform has been found in testis and skin [5, 19, 22, 23].

RORα has been implicated in control of both inflammatory and metabolic signaling. In murine cells, RORα positively regulates *Apoa-I* and *Apoc-III* suggesting its role in lipid metabolism [24, 25]. In human cells RORα has been shown to control expression of *APOA-I*, *ABCA1* and *CYP19A1* metabolic genes [24, 26].

While data supporting RORα control of lipid metabolism appears to be consistent, there is still some disagreement about the exact role of this molecule in control of inflammation. Kopmels *et al*. have demonstrated that macrophages from *staggerer* mice, which have deletion in *Rora* gene, overexpress *Il1b* after LPS stimulation suggesting an anti-inflammatory role for RORα [15]. Moreover, Delerive *et al*. indicated that RORα interferes with the NF-κB signaling pathway. The authors proposed that RORα suppresses inflammatory signaling by inducing IκBα, a negative regulator for NF-κB pathway [27]. Furthermore, *Rora* is involved in production of TNF and IL-6 upon macrophage activation [28] and has been found to play a key role in M1/M2 polarization of murine Kupffer cells that are liver resident macrophages [12]. In the absence of *Rora* Kupffer cells assumed the pro-inflammatory M1 identity and the shift to the M2 profile required RORα activation.

On the other hand, there are several studies providing evidence for the pro-inflammatory role of RORα. For instance, *Rora* deficiency in murine retinas leads to dramatic upregulation of Socs3, key suppressor of cytokine signaling [29]. As a result, expression of inflammatory cytokines in murine retinas has been reduced in *Rora*-deficient mice supporting a case for the pro-inflammatory role for RORα. In addition, Lui *et al*. have recently demonstrated that RORα drives endoplasmic reticulum stress and thus stimulates adipose tissue inflammation [30]. Presence of discordant inflammatory phenotypes in different tissues of the same murine model suggests that the direction of RORα function is modified by cell type-specific factors. This possibility underscores the importance of defining the exact nature of RORα function in human macrophages.

Accumulated evidence about involvement of RORα in both metabolic and inflammatory signaling underscores the importance of understanding the scope, direction and magnitude of its regulatory effects in relevant cell types. In our study, we set out to evaluate the role of RORα in control of inflammatory signaling in human macrophages and to obtain data to address the existing controversies.

## Materials and methods

### Reagents

Penicillin-Streptomycin (P0781), PMA (P8139), βME (M3148), Hank’s balanced salt solution (H9269), DPBS (D8537) and polybrene (107689) were purchased from Sigma Aldrich. DMEM (BE12-604F, Lonza), Trypsin/EDTA (BE17-161E, Biowhittaker), FBS (10270–106, GIBCO) and LPS-EK Ultrapure from the *E*. *coli* K12 strain (tlrl-peklps) from Invivogen. Opti-MEM I Reduced Serum Medium (11058021) from ThermoFisher Scientific and Puromycin (540222) from VWR. BsmB1 restriction enzyme (R0580), T4 DNA ligase (M0202) and T4 DNA ligase reaction buffer (B0202) were from New England Biolabs. PureYield Plasmid Miniprep System (A1222) was from Promega.

### Cells

THP-1 human monocytic cell line (ATCC, TIB-202) maintained in RPMI 1640 (A10491-01, GIBCO) medium supplemented with 10% FBS, 100 U/ml penicillin, 0.1 mg/ml streptomycin and 0.05 mM βME at 37°C in a humidified atmosphere with 5% CO_2_. THP-1 monocytes were differentiated into macrophages using 50 ng/ml PMA for 48 hours and then rested in PMA-free medium for an additional 24 hours before use.

HEK 293T cell line (ATCC CRL-3216) was cultured in DMEM with 10% FBS and 100 U/ml penicillin, 0.1 mg/ml streptomycin at 37°C in a humidified atmosphere with 8% CO_2_. Cells were detached using trypsin and split every 3 days.

### Generation of *RORA kno*ckout THP-1 cell line

Three *RORA* sgRNAs targeting exon 4 and 5 were designed using the Broad Institute sgRNA design tool (https://portals.broadinstitute.org/gpp/public/analysis-tools/sgrna-design) (targets: exon 4: 5՛-ACCATCTCGAGACATCCCTA-3՛, exon 5: 5՛-AGTTGGGGAAGTCTCGCCGT-3՛ and 5՛-GACGCCCACCTACAACATCT-3՛). Oligonucleotides were synthesized and cloned into lentiCRISPRv2 vector (52961, Addgene) individually according to oligo cloning protocol for LentiCRISPRv2 [31, 32]. Three targeting clones were validated by sequencing. Lentiviral particles were then generated according to Addgene protocol (http://www.addgene.org/tools/protocols/plko/#E). In brief, 2.5×10^6^ HEK 293T cells were seeded in 10 cm dishes. Cells were transfected the day after seeding with 2.4 μg of the psPAX2 plasmid (12260, Addgene), 1.2 μg of the pCMV-VSV-G plasmid (8454, Addgene) and 2.4 μg of the lentiCRISPRv2-RORα sgRNA plasmids (0.8 μg of each construct) using GeneJuice transfection reagent (70967, Merck). Media was refreshed 12–15 hours post-transfection. Virus-containing supernatant was collected at day 4 and 5 and stored at -20°C for further use.

0.5–1×10^6^ THP-1 cells in 0.5 ml media were transduced with 1.5 ml virus-containing supernatant using 6 μg/ml of polybrene. Cells were incubated overnight and resuspended in fresh media containing 2 μg/ml Puromycin. After 7 days of selection, cells were subjected to clonal expansion and clones were obtained 40–50 days post-transduction. Presence of mutations in the selected clone was confirmed by sequencing (S1A Fig in S1 File).

THP-1 sgRenilla cells were obtained from Dr. Richard Kumaran Kandasamy to be used as controls. These cells were stably transduced with LentiCRISPRv2 viral construct carrying sgRNA targeting firefly *Renilla* gene that does not exist in human genome.

### RNA isolation and quantitative real-time PCR

Total cellular RNA was isolated using QIAcube instrument and RNeasy Mini kit (74106) with DNAse I digestion (79254) (all Qiagen) according to the manufacturer’s instructions. Relative quantifications were performed by quantitative real-time RT-PCR using StepOnePlus PCR system, TaqMan probes and TaqMan RNA-to-CT 1-Step Kit (4392938, ThermoFisher Scientific) according to manufacturer’s protocol. Probes: TNF, Hs00174128_m1; IL6, Hs00985639_m1; IL1B, Hs00174097_m1; GAPDH, Hs99999905_m1 and TREM1, Hs00218624_m1. Gene expression levels were normalized to levels of GAPDH that was used as an endogenous control and relative expression values were calculated as fold induction over controls.

### Measurements of cytokine levels (multiplex)

Levels of cytokines secreted in the cell supernatants were analyzed using the Bio-Plex human cytokine 5-plex panel (Bio-Rad Laboratories, Hercules, CA) containing the following analytics: TNF, IL-1β, IL-1α, IL-6 and IL-18. The analysis was performed according to manufacturer instructions.

### LDH assay

1x10^5^ cells per well were differentiated in 96-well plate. Spontaneous and LPS induced LDH release was measured using Pierce LDH Cytotoxicity Assay Kit (88953) according to manufacturer’s protocol. Proportion of dead cells was determined by calculating the ratio of the LDH amounts in the supernatant to the amount of total LDH after cell lysis.

### Western blot

Protein extracts from cell lysates were separated on NuPAGE 4–12% Bis-Tris Protein Gel (NP0301BOX, Invitrogen) according to manufacturer’s instruction. Transfer of separated proteins to nitrocellulose membrane was carried out using iBlot 2 Gel Transfer Device (IB21001, ThermoFisher Scientific). Membranes were blocked in 5% BSA/TBST and stained using human IL-1beta/IL-1F2 (AF-201, R&D systems), ROR alpha (NBP2-24493, NOVUS biologicals), beta tubulin (ab6046, Abcam) and PCNA (FL-261, sc-7907, Santa Cruz Biotechnology) antibodies. Blots were then developed with SuperSignal West Femto maximum sensitivity substrate (34096, Pierce) and imaged with a LI-COR Odyssey Fc system.

### RNAseq analysis

Total RNA was isolated from duplicate differentiated knockout and control cell lines. RNA quality control was performed using Agilent 2100 Bioanalyzer System. Libraries were prepared at Novogene (Hong Kong) and sequenced on Illumina platform (Novogene, Hong Kong) as 150 bp paired-end reads with Q30≥90% (S1 Table in S2 File). Short sequence reads were quasi-mapped to GRCh38 human transcriptome using *salmon* v0.11.3 transcript quantification tool [33]. Transcript reads length scaled and summarized at the gene levels using *tximport* tool and Ensemble Release 94 Human genes (GRCh38.p12) annotation database. Obtained data object was fed into the *limma-voom* pipeline [34]. Low abundance transcripts were filtered out (>10TPM/sample/ >15TPM group). After fitting, differences in expression was evaluated using *treat* function and differentially expressed genes were defined as those with LFC>1 and adjusted p value of <0.05. *goana* and *kegga* functions were used for GO term enrichment and KEGG pathway analysis respectively. Heatmaps were created using *ComplexHeatmaps* Bioconductor package [35]. RNAseq data have been deposited in the ArrayExpress database at EMBL-EBI (www.ebi.ac.uk/arrayexpress) under accession number E-MTAB-7295.

### Statistical analysis

Statistical analysis for experiments consisting of minimum three independent biological replicates was done using GraphPad Prism software package. Significance of pairwise comparisons was evaluated using two-tailed student *t* test. Significance of differences observed in the timecourse experiments was determined using ANOVA two-tailed test with the post hoc Bonferroni correction. P values below 0.05 were considered as statistically significant. Column figures are presented as means ± SD.

## Results

### RORα is a negative regulator of inflammation in THP-1 cells

**Fig 1:**
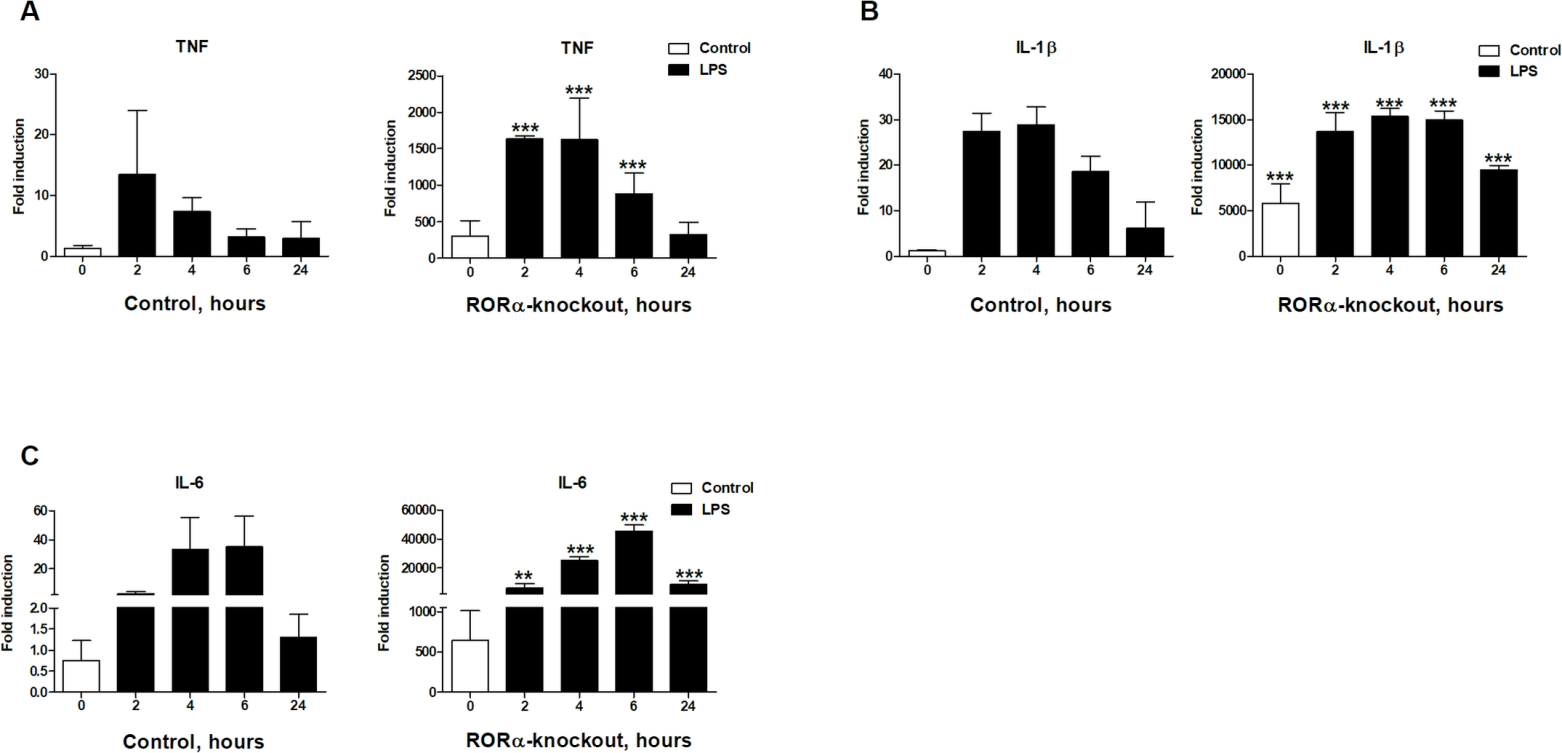
Cells lacking *RORA* have elevated inflammatory state. Deletion of *RORA* led to increase in basal and LPS-induced cytokine mRNA levels. **A.** *TNF* transcript levels are elevated approximately 300 fold in differentiated knockout cells, **B.** *IL1B* mRNA were nearly 5000 fold and **C.** *IL-6* almost 700 fold higher than in the control cell line. 2.5×10^5^ cells in 24-well plates were differentiated using 50 ng/ml PMA for 48 hours. Stimulation was performed after 24 hours of rest in PMA-free media with 10 ng/ml LPS for 2, 4, 6 and 24 hours. Inflammatory cytokines expression levels were determined using qRT-PCR of RNAs isolated from cell lysates. Graphs are representatives for at least three independent experiments. Statistical significance was calculated using ANOVA test. Stars indicate significance of differences between control and knockout cells after post hoc Bonferroni correction.

Previously the inflammatory function of RORα has almost exclusively been studied using murine models [15, 36]. To test which of the observations made in the model system translate to human cells, we chose to determine the role of RORα in control of inflammatory signaling in human macrophages. To achieve this we analyzed the effect of deleting *RORA* gene in widely used THP-1 monocytic cell line [37]. We used lentiCRISPRv2 lentiviral delivery system [31, 32] to simultaneously introduce guide RNAs targeting *RORA* gene and Cas9 nuclease into THP-1 cells. Following selection of transduced cells and single cell cloning, we confirmed deletion of both *RORA* alleles by sequencing (S1A Fig in S1 File). When compared to control cell lines carrying irrelevant *renilla* guide RNA, we found that deletion of *RORA* in THP-1 cells made a substantial impact on their inflammatory state (Fig 1). Even in the absence of stimulation we saw that expression levels of selected inflammatory cytokines were dramatically higher in *RORA*-KO cells compared to THP-1 sgRenilla control cells. Exposure of knockout cells to 10 ng/ml of K12 LPS (Invivogen) further induced mRNA levels of these cytokines and the induced levels remained significantly higher than those in the control cells at most tested time points post-stimulation. This observation of increased inflammatory cytokine production in cells lacking *RORA* supports the anti-inflammatory role of this gene.

### *RORA* deletion drives secretion of pro-inflammatory cytokines

**Fig 2:**
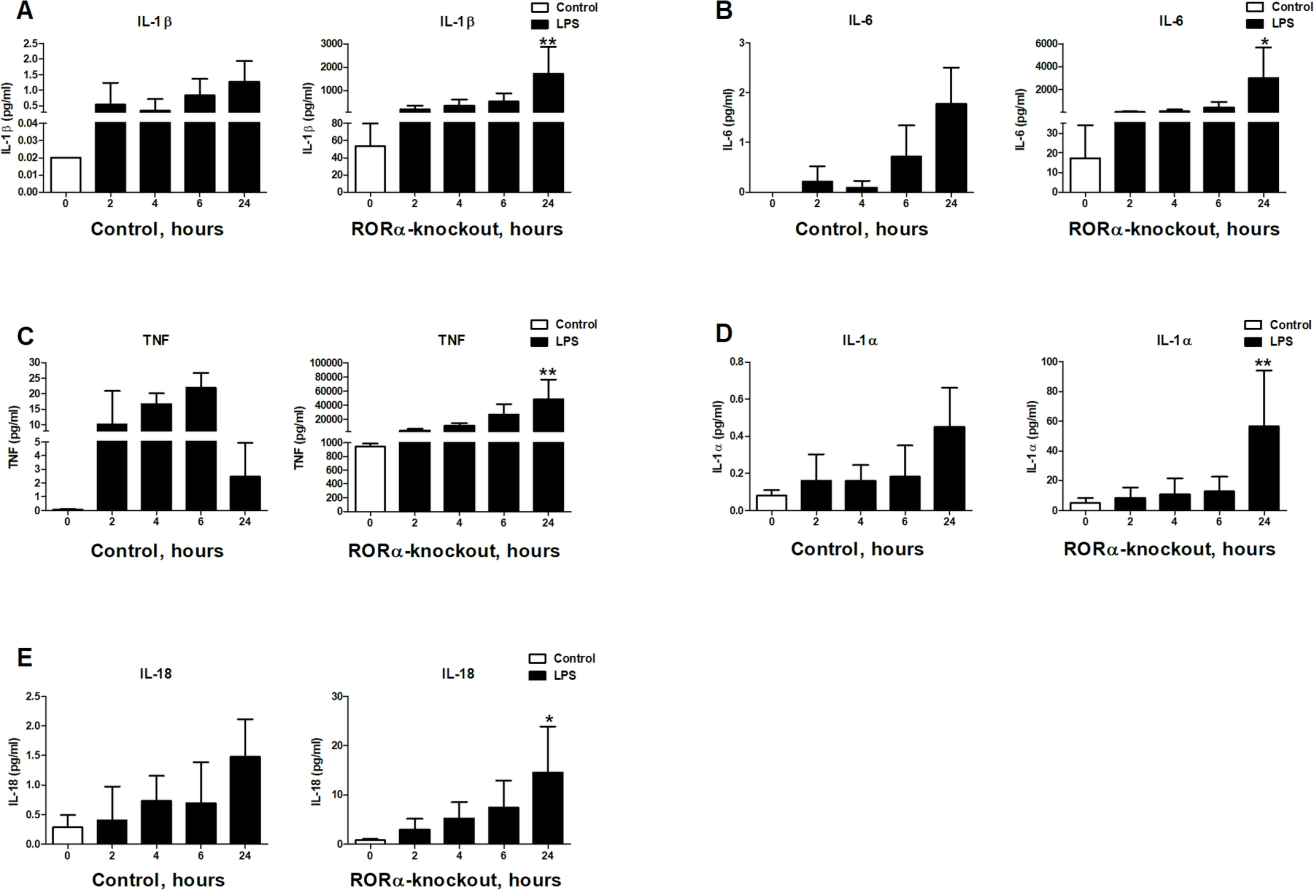
Cytokine secretion by *RORA* knockout cells. Deletion of *RORA* led to increase in basal and LPS-induced cytokine secretion. **A.** IL-1β has the biggest increase of approximately 2500 folds, **B.** IL-6 levels increases nearly 15 folds, **C.** TNF levels increases almost 900 folds, **D.** IL-1α levels increases approximately 50 folds and **E.** IL-18 levels increases 5 folds in deletion cells. 2.5×10^5^ cells in 24-well plates were differentiated using 50 ng/ml PMA for 48 hours. Stimulation was performed after 24 hours of rest in PMA-free media with 10 ng/ml LPS for 2, 4, 6 and 24 hours. Cytokines quantification was performed using multiplex cytokine assay on the supernatant. Graphs are representatives for at least three independent experiments. Statistical analysis was calculated using ANOVA test. Stars indicate significance of differences between control and knockout cells after post hoc Bonferroni correction.

To determine if observed increase in mRNA levels of inflammatory cytokines results in increased protein production, we analyzed cytokine accumulation in supernatants of differentiated THP-1 sgRenilla control and *RORA*-deletion cell lines over 24 hour period of time. We used Bio-Plex assay to measure levels of TNF, IL-1β, IL-18, IL-1α and IL-6 proteins before and after stimulation with 10 ng/ml LPS. We observed that even in unstimulated cells there were higher levels of all of the examined cytokines. Stimulation with LPS further increased secretion of these cytokines. Therefore, our data indicate that even though *RORA*-deletion cells are in the constitutively elevated inflammatory state, they are still fully capable to sense and respond to LPS (Fig 2).

Presence of IL-1β in supernatants of unstimulated cells would suggest that *RORA*-deletion cells either actively secrete this cytokine or are dying and releasing abundant pro-IL-1β into supernatants [38]. To differentiate between these two scenarios, we tested if *RORA* knockout cells display higher rate of spontaneous cell death when compared to THP-1 sgRenilla control cells. We measured LDH levels in the supernatants of deletion and control cells to assess the extent of spontaneous and LPS induced cell death. We found that cells lacking *RORA* had a similar rate of spontaneous cell death as our control cell lines (S2 Fig in S1 File). LPS stimulation resulted in slight increase in cell death of *RORA*-mutant cells but the majority of cells appeared to be alive.

### *RORA*-deletion cells constitutively produce pro-IL-1β

**Fig 3:**
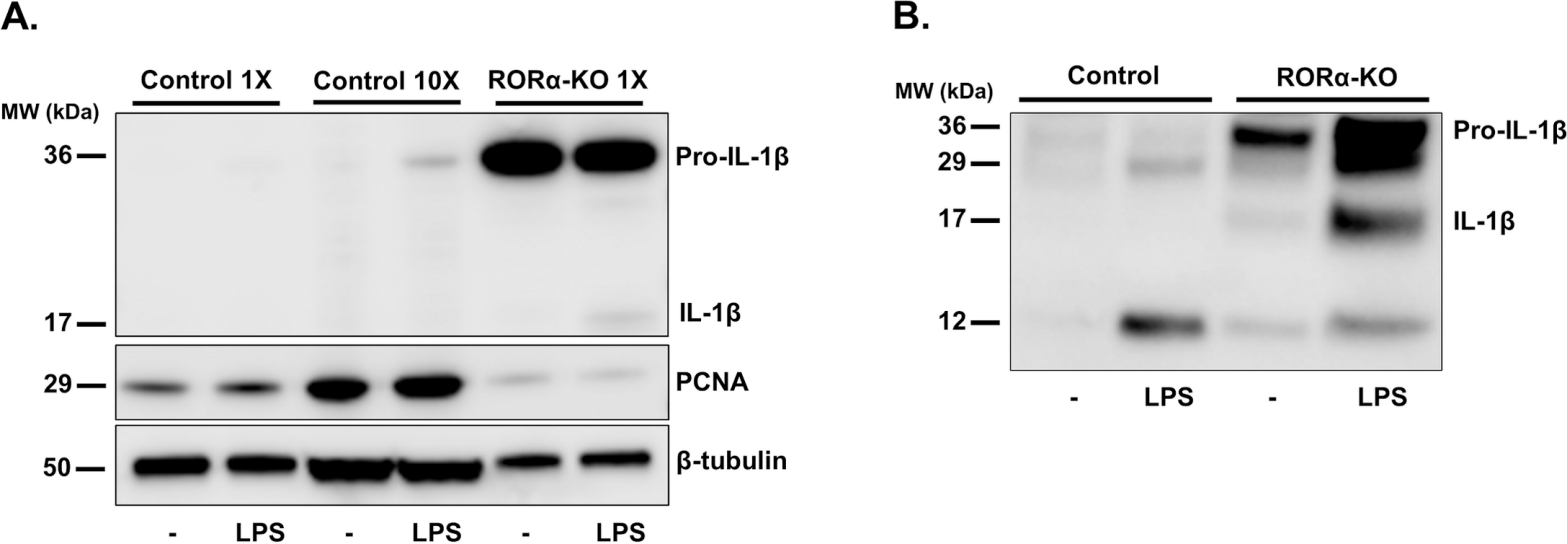
*RORA* knockout cells produce IL-1β. **A.** Western blot analysis of total cell lysates of *RORA*-deletion cells reveals accumulation of pro-IL-1β even in the absence of LPS stimulation. Pro-IL-1β was not detectable in LPS-stimulated lysates from the equivalent number of control cells and was detectable only in 10-fold excess of lysates. PCNA and beta Tubulin were used as loading controls. **B.** Western blot of supernatants after 24 hours incubation with or without 10 ng/ml LPS. *RORA*-deletion strains accumulate substantial amounts of pro-IL-1β in the supernatants even in the absence of LPS stimulation. Addition of LPS stimulated secretion of some mature IL-1β from *RORA*-KO cells.

We observed that *RORA*-deletion cells have substantial amounts of IL-1β in supernatants even in the absence of stimulation (Fig 2). IL-1β production requires two signals consisting of transcriptional activation and inflammasome-mediated post-translational proteolytic processing of the pro-peptide. Our observation suggests that both signals could be activated in *RORA*-deletion cells. As a first step we chose to test if IL-1β pro-peptide is constitutively produced by cells lacking *RORA*. Western blot analysis shown in Fig 3, confirms that *RORA*-knockout cells, unlike THP-1 sgRenilla control cells, produce substantial amounts of pro-IL-1β in the absence of stimulation. Stimulation of control cells with 10 ng/ml of K12 LPS induced production of pro-IL-1β (Fig 3A) and secretion of low levels of IL-1β into supernatants (Fig 2). On the other hand in *RORA* knockouts there was no additional increase in pro-IL-1β production. It appeared though that LPS stimulation induced proteolytic processing of pro-IL-1β as illustrated by appearance of the cleaved IL-1β band (Fig 3A) and secretion of large amounts of IL-1β into supernatants (Fig 2). Western blot analysis of supernatants from control and mutant cells incubated for 24 hours with or without 10 ng/ml LPS revealed that *RORA*-deletion cells accumulate substantial amounts of pro-IL-1β (Fig 3B). In the presence of LPS we detected some mature IL-1β in supernatants from *RORA*-KO cells. We also observed prominent 29 kDa band that has previously been described as caspase-1 dependent inactive form of IL-1β [39]. 12 kDa is an apparent product of IL-1β degradation by extracellular proteases.

### *TREM1* is upregulated in mutant cells

**Fig 4:**
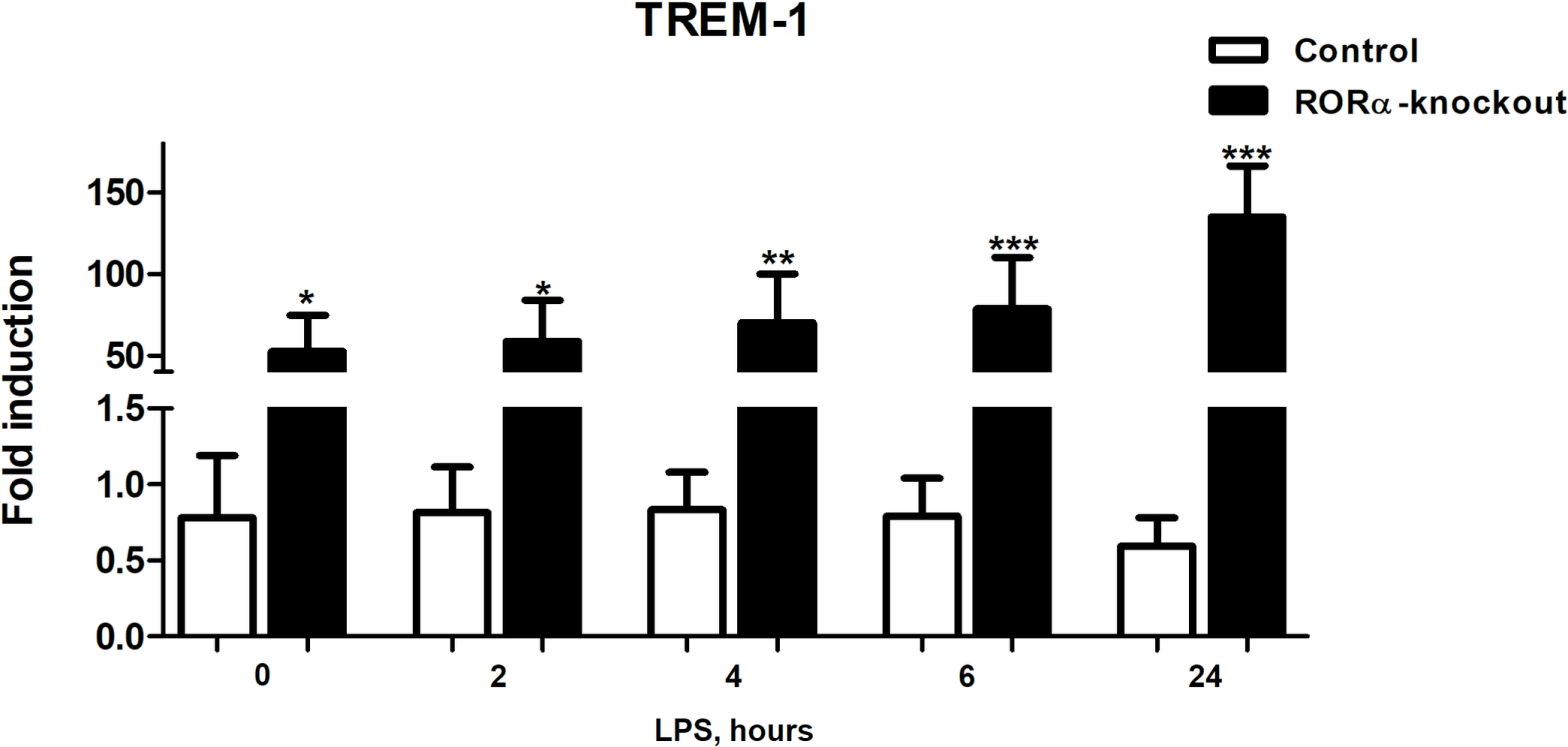
Cells lacking *RORA* have elevated levels of *TREM1* mRNA. *TREM1* was expressed at significantly higher levels in untreated *RORA*-deletion cells when compared to THP-1 sgRenilla control cells. *TREM1* expression was not significantly affected by treatment with LPS. 2.5×10^5^ cells in 24-well plates were differentiated using 50 ng/ml PMA for 48 hours. After 24 hours of rest in PMA-free media, *TREM1* expression level was determined using qRT-PCR for RNAs isolated from cell lysates. Statistical significance was calculated using ANOVA test. Stars indicate significance of differences between control and knockout cells.

*RORA* knockout cells spontaneously produced substantial amounts of TNF, IL-6 and IL-1β. Production of these cytokines is characteristic of the classically activated M1 macrophages [12, 40]. Triggering Receptors Expressed on Myeloid cells (TREM1), is a cell surface receptor that acts to amplify acute and chronic inflammation [41]. Furthermore, activation of TREM1 drives secretion of M1 inflammatory cytokines, such as TNF, IL-1β and IL-6. It has been proposed that increased expression of *TREM1* drives the inflammatory transformation of macrophages. We therefore chose to test if *TREM1* mRNA expression is altered in *RORA* knockout cells. We found that *TREM1* expression is significantly upregulated in mutant cells (Fig 4). Notably, the levels of *TREM1* expression were not significantly impacted by LPS stimulation, suggesting that its expression is not modulated by the levels of secreted inflammatory cytokines due to LPS-induced response.

### THP-1 cells lacking *RORA* have altered transcriptome

**Fig 5:**
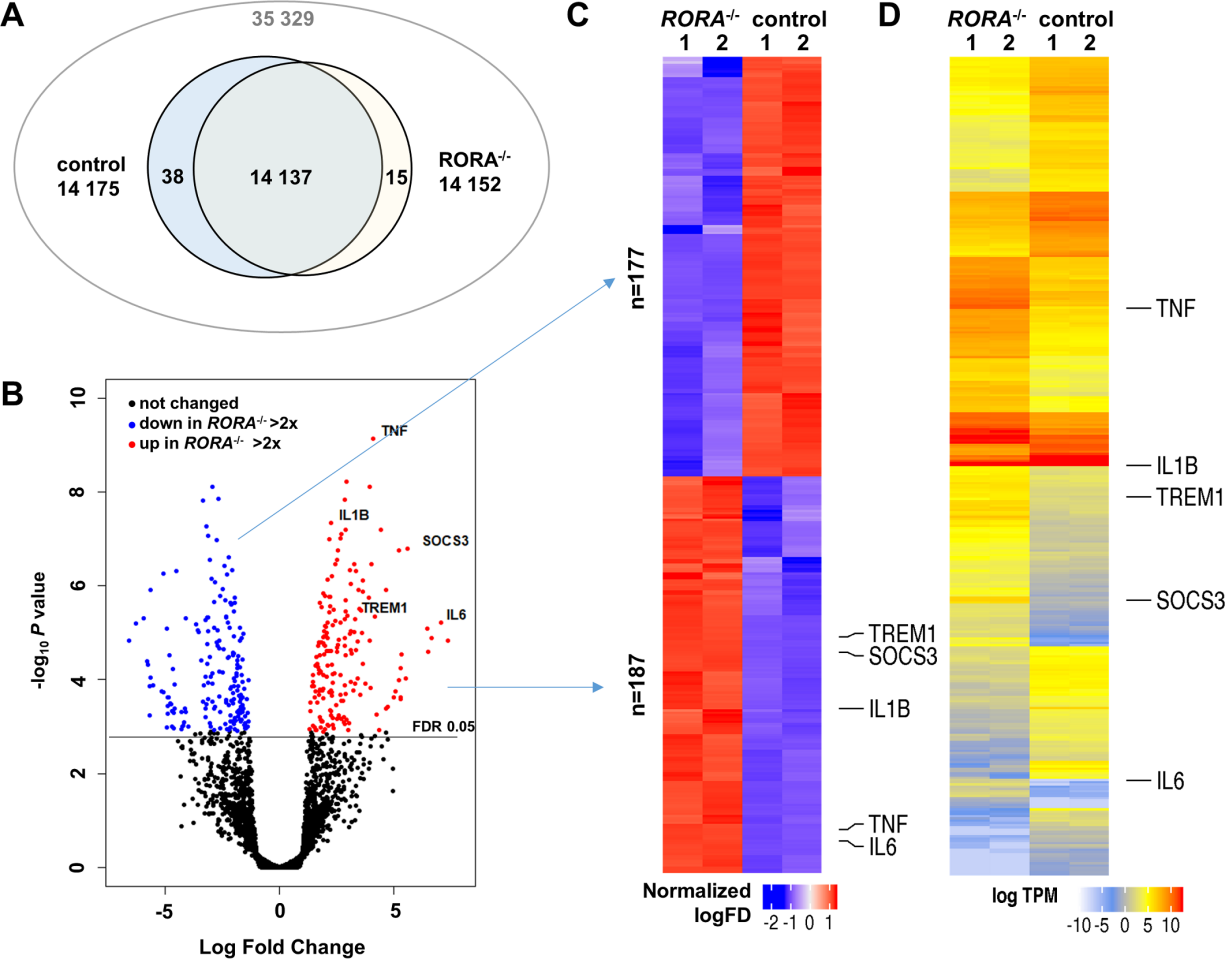
Deletion of RORA changes gene expression in differentiated THP-1 cells. **A.** Reads mapped to the human transcriptome were summarized for 35329 annotated genes. After filtering out genes with low read counts the remaining 14190 genes were used in differential expression analysis. **B.** Volcano plot shows distribution of raw *p*-values of identified 187 genes downregulated >2 fold in RORA-/- cells (blue) and 177 2-fold upregulated genes (red). **C.** Heatmap of 364 identified differentially expressed genes clustered by the overall expression levels. **D.** Heatmap of differentially expressed genes clustered by normalized read counts.

RORα has been proposed to play a global regulatory role in control of human immune cell gene expression. It has been suggested that RORα binds to genomic regions near transcription start sites of as many as 3000 genes in THP-1 cells [42]. To determine the extent of the transcriptome remodeling induced by the deletion of *RORA* we analyzed gene expression in unstimulated differentiated THP-1 cell lines by RNAseq. Sequence reads were mapped to human transcriptome and transcript reads were aggregated for gene level differential expression analysis. A total of 14190 genes were found to be expressed in the analyzed samples using >10 Counts Per Million (CPM) per sample or >15 CPM per group cutoff. Most of the mapped genes (14 137) had reads in both control and knockout samples. 15 genes, including *IL6*, had over the threshold number of reads only in knockout cells and 38 only in control lines (Fig 5A). We found that there was a relatively small set of genes, consisting of 364 transcripts, that were differentially expressed more than 2-fold when using False Discovery Rate (FDR) adjusted *p*<0.05 cutoff for significance (Fig 5B and S2 Table in S2 File) in mutant cells compared to THP-1 sgRenilla control cells. Differentially expressed genes were nearly evenly split, with 187 that were upregulated and 177 downregulated in the knockout cells (Fig 5C). In control cells *IL6* and *SOCS3* transcripts were nearly absent (Fig 5D), while *IL1B* messages were quite abundant.

**Fig 6:**
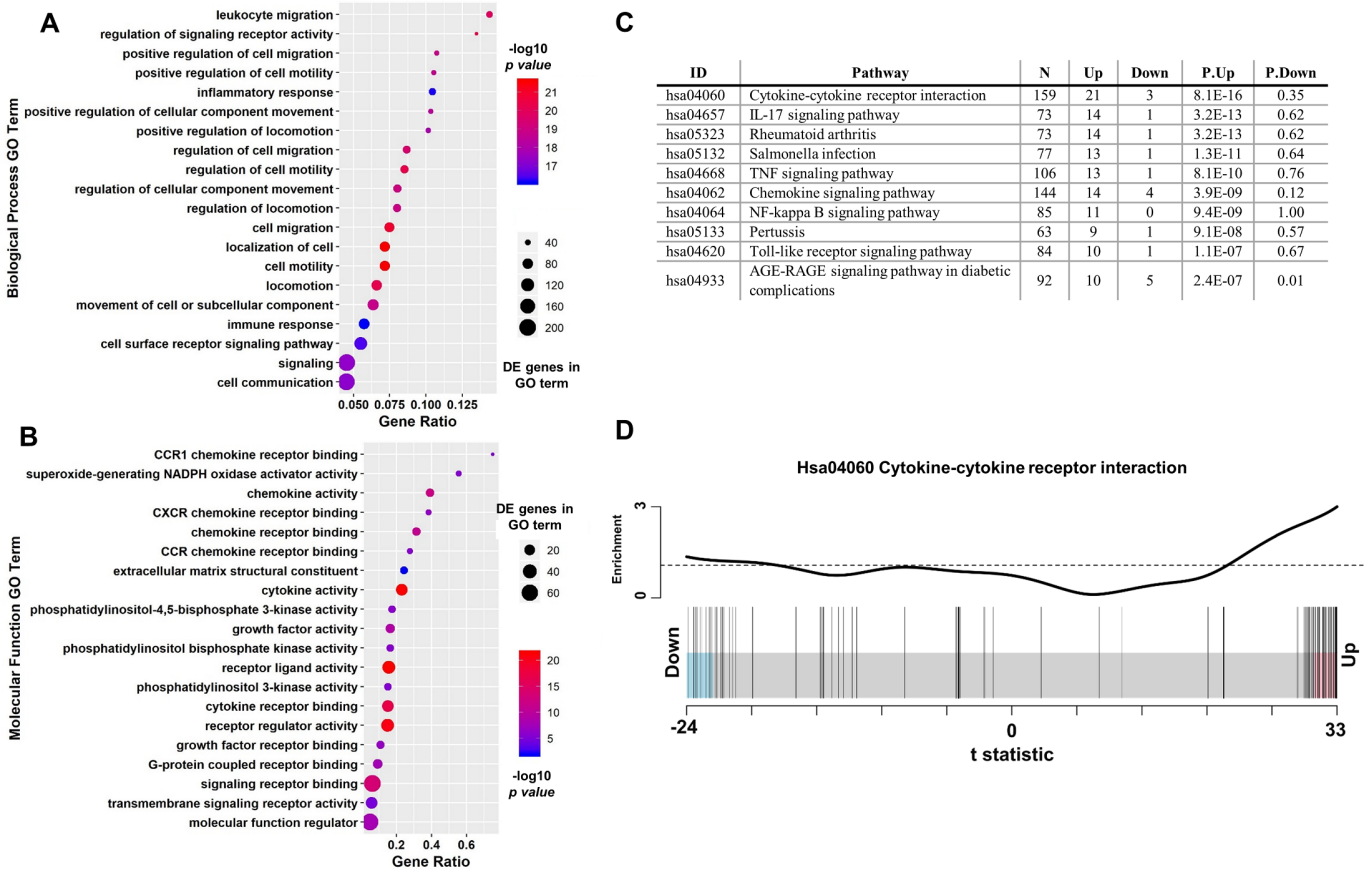
Inflammatory signaling GO terms and pathways are overrepresented by genes upregulated in RORA deletion cells. **A.** Top 20 Biological Process GO terms enriched among differentially expressed genes describe inflammatory signaling mediated processes. **B.** Similar overrepresentation of inflammation related functions was found among the Top 20 Molecular Function GO terms. The ratio of identified differentially expressed genes to the total number of genes within each term is shown on the x-axis. **C.** Top 10 KEGG pathways enriched by genes upregulated in the RORA knockout cells are all involved in mediating inflammatory signaling. **D.** Barcode plot illustrating overrepresentation of upregulated (21 out of 24) differentially expressed genes in of the Cytokine-cytokine receptor interaction pathway. Only 3 downregulated genes were represented in this pathway.

Gene Ontology terms describing inflammatory signaling were the most enriched by the genes that are differentially expressed in differentiated *RORA* knockout THP-1 cells within both the Biological Process (Fig 6A) and Molecular Function (Fig 6B) domains. In fact all 20 of the top Biological Process terms describe inflammatory and cytokine/chemokine mediated processes. The global impact of *RORA* deletion on inflammatory signaling was further confirmed by the KEGG pathway enrichment analysis. It revealed that mostly inflammatory signaling pathways are significantly impacted by the deletion of *RORA* (Fig 6C). In agreement with our initial observations, cytokine-cytokine receptor interaction and TNF signaling pathways were among the most significantly enriched by genes upregulated in the *RORA* knockout. Within the top enriched KEGG *hsa04060*:*Cytokine-cytokine receptor interaction* pathway 21 out of the 24 differentially expressed genes were upregulated in deletion cells (Fig 6D). Consistent with the documented direct involvement of *RORA* in regulation of cellular metabolism [26], several metabolic pathways were enriched by genes downregulated in the knockout THP-1 macrophages (S3 Table in S2 File). Taken together our data suggests that RORα is responsible for maintenance of the inflammatory baseline in human macrophages and in its absence cells enter the constitutive hyper-inflammatory state.

## Discussion

The main finding of our study is that RORα controls the inflammatory state of human macrophages. *RORA* deletion resulted in constitutive activation of transcription for a subset of NF-кB regulated genes including *TNF* and *IL1B*. Furthermore, our subsequent RNAseq analysis revealed significant changes in the transcriptome of the *RORA*-deletion cells that resembled the classically activated M1 macrophages.

There is still a disagreement about how RORα impacts inflammatory signaling [15, 28, 29, 43]. Our work was designed to address some of the outstanding issues regarding RORα function. We approached this question by deleting both alleles of *RORA* in human monocyte-like THP-1 cell line. Our deletion in exons 4 and 5 would result in a frameshift generating a premature stop codon at amino acid 143 (S1A Fig in S1 File). We found that deletion of *RORA* resulted in elevated expression of pro-inflammatory cytokines in both undifferentiated and differentiated mutant cells (Fig 1 and S3 Fig in S1 File). This observation supports the anti-inflammatory function of RORα in human macrophage-like cells. Interestingly, even though the basal expression levels of *TNF*, *IL-1β* and *IL-6* are higher in mutant cells, in response to LPS stimulation the magnitude of induction is similar in both mutant and control cells. This suggests that lack of RORα did not interfere with response to LPS.

*TNF* is one of the key early genes expressed during inflammatory response and reaches the highest level of expression at 2 hours post-stimulation in control cells. Levels of its mRNA expression begin to decrease at later time points. In mutant cells, *TNF* mRNA levels rose to their peak at 2 hours but stayed at that level past 4 hours after LPS stimulation. This suggests that *RORA* deletion cells lack means to downregulate NF-кB signaling. This hypothesis is consistent with the observed pattern of *IL1B* expression (Fig 1) that also persists for extended period of time.

Release of mature IL-1β is a hallmark of pyroptosis and therefore we tested if deletion cells display high rate of spontaneous cell death. We found no significant increase in release of LDH over 24 hours incubation of unstimulated cells. Considering the amounts of pro-IL-1β produced by cells lacking *RORA* (Fig 3A), this rate of cell death will account for the presence of pro-IL-1β in the supernatants (Fig 3B). Addition of LPS led to slight increase in cell death of *RORA* mutants but the majority of cells appeared to be alive and could produce mature 17 kDa IL-1β (Fig 3B). This observation is consistent with a recently published observation of living macrophages being able to secrete IL-1β [44].

Our data suggests that the consequences of the absence of *RORA* are not limited to changes in inflammatory cytokine expression alone. Detection of pro-inflammatory cytokines such as IL-18 and IL-1β in the supernatant in the absence of notable differences in cell death suggests that both transcriptional activation and post-translational processing could have been triggered in mutant cells. It appears that LPS stimulation accelerates proteolytic processing of pro-IL-1β as indicated by apparent a mature IL-1β 17 kDa band inside LPS-induced *RORA* deletion cells (Fig 3) and dramatic increase of secreted IL-1β in supernatants (Fig 2). Our interpretation is that in the absence of *RORA* cells are in the constitutive “primed” state and exposure of these cells to LPS promotes inflammasome activation. Overall, these data imply that *RORA* deletion leads to hyper-inflammatory state and that LPS stimulation triggers pro-IL-1β cleavage to produce mature form.

Cells lacking *RORA* produce a set of cytokines consistent with that produced by conventionally activated M1 macrophages [12]. It has recently become apparent that TREM1 is one of cell surface regulators of the cytokine production amplification [41] and it is involved in driving M1 polarization. We found *TREM1* expression to be highly upregulated in deletion cells. Interestingly, the levels of *TREM1* expression were not further modified by stimulation with LPS. This suggests a possibility that *TREM1* might be negatively regulated by *RORA* on the transcriptional levels. While analysis of potential RORα target genes in THP-1 cells [42] did not reveal putative binding sites in proximity of *TREM1*, the regulatory relationship between *RORA* and *TREM1* needs to be explored further.

The exact set of markers that differentiate between human M1 and M2 macrophages is a subject of continued debate. Recently it has been suggested that high expression levels of *S100A8*/*S100A9* and *CLEC5A* can be used to define classically activated M1 human macrophages [45, 46]. We found that these genes are also upregulated in *RORA*-deletion cells (S2 Table in S2 File) further supporting our view of RORα as a molecule that defines the inflammatory state of human macrophages.

Macrophages are thought to express *RORA1* and *RORA4* isoforms [47]. Our deletions are in exons 4 and 5 that are shared by all isoforms, therefore eliminating production of all versions of the protein. It is therefore possible that we see the effect of one of the 2 existing isoforms in human macrophages. In future experiments it would therefore be interesting to establish if reintroduction of a specific *RORA* isoform could reverse all or some of the transcriptional changes induced by *RORA* deletion.

Deletion of *RORA* has remodeled the transcriptome of differentiated THP-1 macrophages as illustrated by our RNAseq data (Fig 5). We found that large set of inflammatory cytokines and chemokines were upregulated, further supporting our hypothesis that deletion of *RORA* drives macrophages into classically activated M1-like state. Interestingly the anti-inflammatory role of RORα has been attributed to its repression of the negative regulator of the cytokine signaling SOCS3 [29]. Our data supports RORα involvement in control of *SOCS3* expression. In our RNAseq dataset *SOCS3* was one of the most upregulated genes (log FC 5.63) in *RORA*-deletion cells (Fig 5 and S2 Table in S2 File). However, it appears that in macrophages lacking *RORA*, the SOCS3 anti-inflammatory function is overridden and the cells produce massive amounts of inflammatory cytokines. In this respect our data is consistent with observations made in *sg* mouse peritoneal macrophages [15].

In our study we have primarily used PMA-differentiated THP-1 monocyte-like cell line. This widely used model resembles macrophages differentiated from circulatory monocytes that were recruited in response to inflammatory signals. Considering the diversity of human macrophage populations this limits the extent to which our observations can be applied to predicting behavior of primary cells. Nevertheless our data provides initial evidence of the important role of RORα in human macrophage function.

Our work for the first time demonstrated a key role of human RORα in setting the inflammatory state of human macrophages. This data is consistent with original observations made in murine macrophages from spontaneous *sg* mutant lines. Our data therefore provides evidence that helps resolve some of the discrepancies found in the current literature. Furthermore our work provides foundation for future studies aimed at defining function of specific RORα isoforms.

## Supporting information

S1 File Supplementary figures. (PDF)

S2 File Supplementary tables. (PDF)
