# Supplementary material for: RORα controls inflammatory state of human macrophages
Source: PLoS One. 2018 Nov 28;13(11):e0207374. doi: 10.1371/journal.pone.0207374 (PMC6261595; doi:10.1371/journal.pone.0207374)
Supplement: S1 File — (PDF) [file pone.0207374.s001.pdf]

Supplementary Figures:

S1 Figure:

A

Exon 4 alignment with Chromosome 15:

```
         C  L  A  V   G  C  L  E  M  L  *
00000001 tgccttgccgta.ggatgtctcgagatggtgagctctcacagcttgag  RORA KO
<<<<<<<< |||||||||||| |||||||||||||||||||||||||||||||||||
60514643 tgccttgccgtagggatgtctcgagatggtgagctctcacagcttgag  hg38_genome
         C  L  A  V  G  M  S  R  D  A  V  K  F  G  R  M
```

Exon 5 alignment with Chromosome 15:

```
00000251 cgctgacgcccacctacaaca.....gccaacgggctgacggaacttcac RORA KO
<<<<<<<< |||||||||||||||||||||     ||||||||||||||||||||||||
60511504 cgctgacgcccacctacaacatctcggccaacgggctgacggaacttcac hg38_genome
```

B

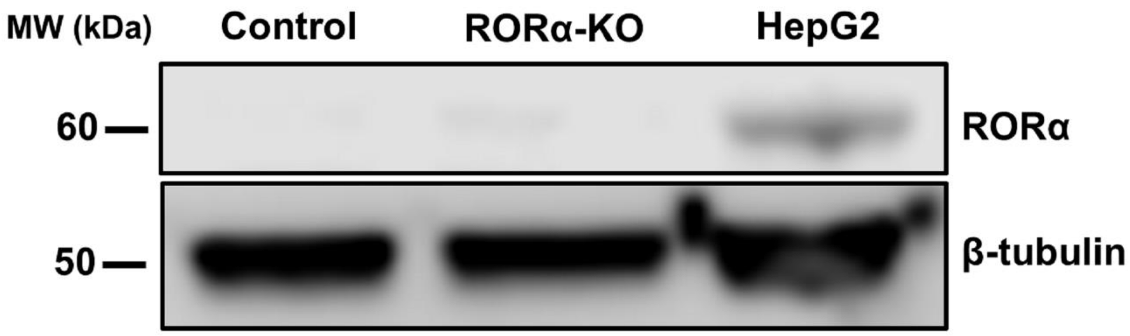

S1 Fig. *RORA* gRNAs introduced lesions in the genome of THP-1 cells. Sequencing of exons 4 and 5 of single cell clones revealed lesions in these two exons. A) 2 out of 3 gRNAs (boxed) used to transduce THP-1 cells introduced deletions 3 bp upstream of the Cas9 NGG PAM site. The single base pair deletion in exon 4 results in a frameshift generating a stop codon 5 amino acids downstream. **B)** RORα protein is not detectable in either control or *RORA*-KO cells, most likely due to low *RORA* expression levels in these cells. HepG2 cell line is used as positive control.

S2 Figure:

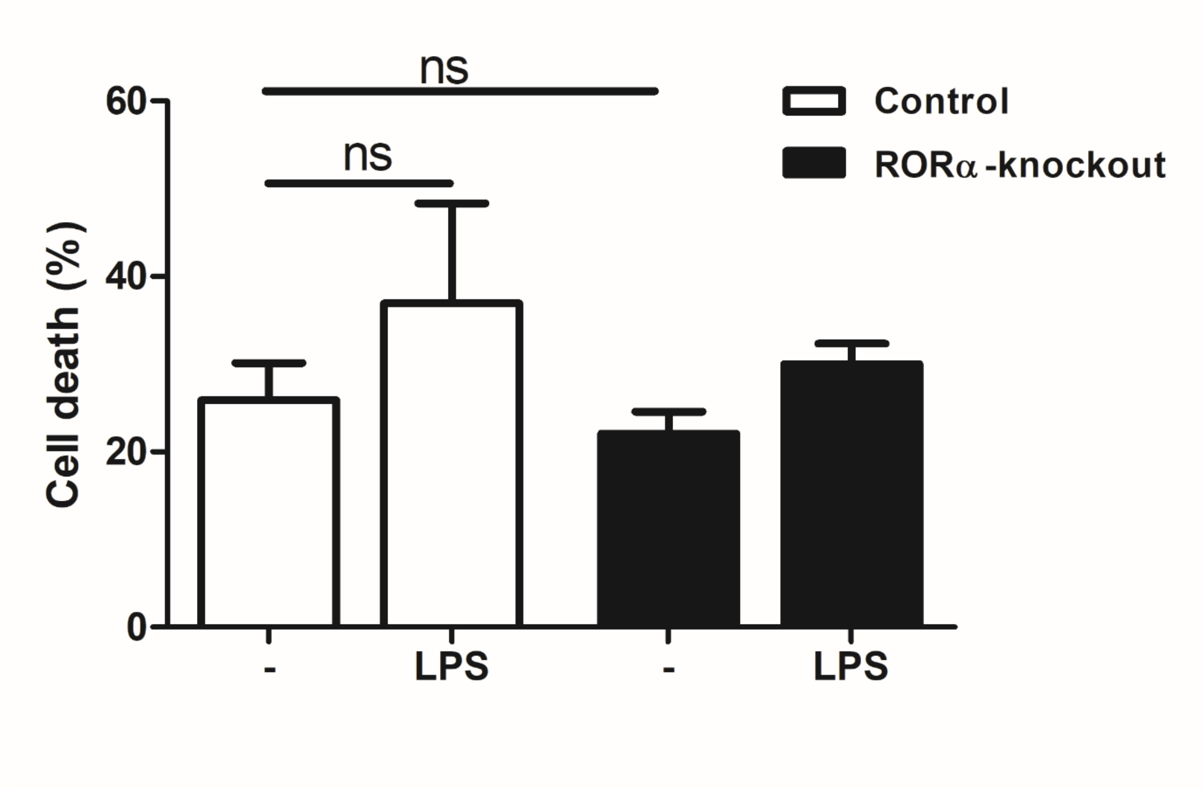

S2 Fig. *RORA*-KO cells show similar viability to control cells. Both control and deletion cell lines had a comparable rate of cell death over 24 hours period of time as measured by LDH release assay. 10^5 cells in 96-well plates were differentiated using 50 ng/ml PMA for 48 hours. Stimulation was performed with 100 ng/ml LPS for 24 hours. Supernatant of unstimulated and LPS-stimulated cells were collected and subjected to LDH release assay.

S3 Figure:

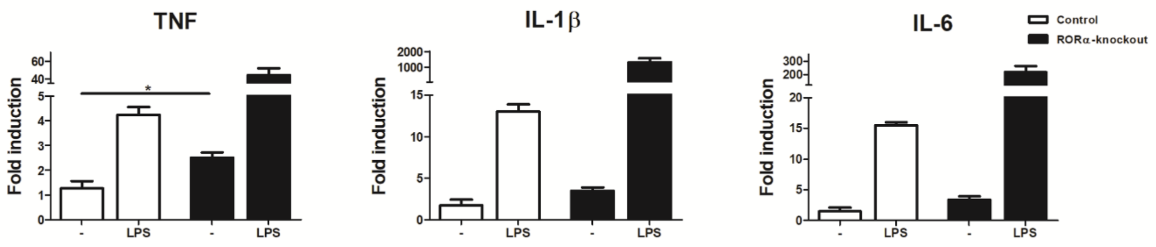

S3 Fig. Expression of inflammatory genes in undifferentiated *ROR*-KO cells. 2.5×10^5 cells in 24-well plates were stimulated with 10 ng/ml LPS for 4 hours. Pro-inflammatory cytokine expression levels were determined using qRT-PCR. Graphs are representatives for at least three independent experiments.

S4 Figure:

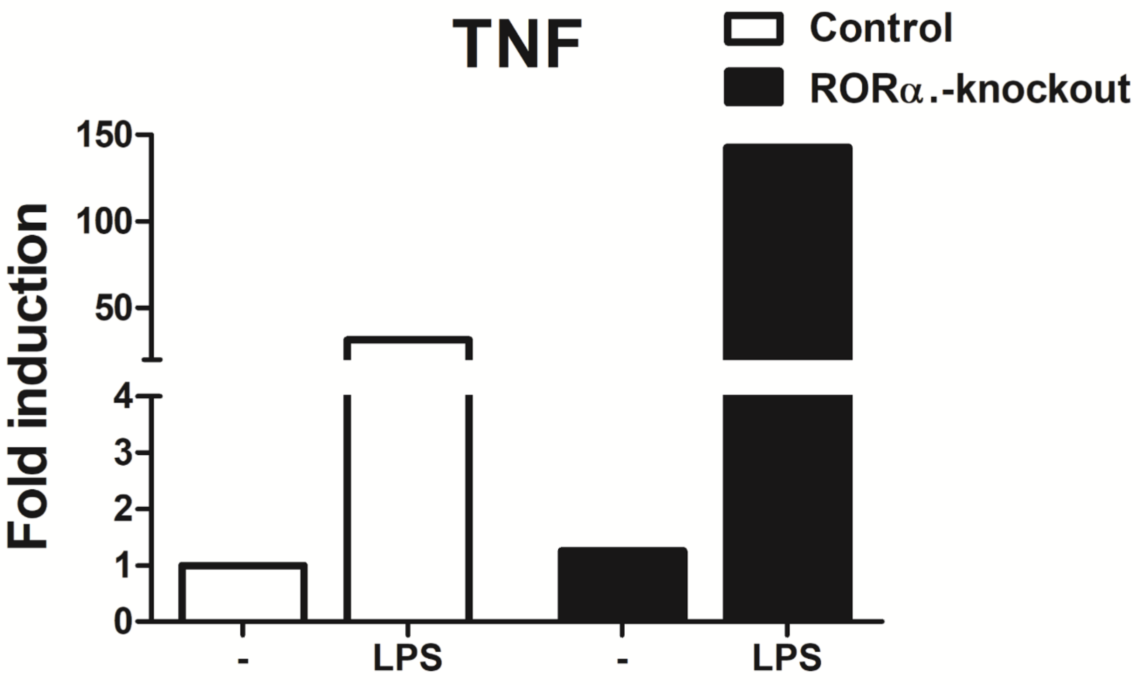

S4 Fig. *TNF* expression is increased in THP-1 cell population transduced with *RORA* sgRNA lentiCRISPRV2 construct.
