# Supplementary material for: RORα controls inflammatory state of human macrophages
Source: PLoS One. 2018 Nov 28;13(11):e0207374. doi: 10.1371/journal.pone.0207374 (PMC6261595; doi:10.1371/journal.pone.0207374)
Supplement: S2 File — (PDF) [file pone.0207374.s002.pdf]

S1 Table. RNASeq Data quality summary

Sample	Raw Reads	Clean Reads	Raw Base(G)	Clean Base(G)	Effective Rate(%)	Error Rate(%)	Q20(%)	Q30(%)	GC Content(%)
Renilla1	30865152	30259274	9.3	9.1	98.04	0.01	97.11	92.91	53.27
Renilla2	29215228	28541024	8.8	8.6	97.69	0.01	97.07	92.83	53.91
RoRa1	26523101	25765191	8.0	7.7	97.14	0.01	97.46	93.66	52.94
RoRa2	27732053	27353567	8.3	8.2	98.64	0.01	97.08	92.85	53.62

S2 Table. List of genes differentially expressed in differentiated *RORA*-deleted THP-1 cells

364 differentially expressed genes grouped according to the direction of changes and sorted by the t value

Gene	logFC	AveExpr	t	P.Value	adj.P.Val
TNF	4.11	6.7754	32.82	7.30E-10	1.04E-05
MMP1	2.94	7.4368	24.93	5.88E-09	2.77E-05
CXCL1	3.95	5.3925	24.06	7.82E-09	2.77E-05
GIMAP8	2.86	7.0602	22.12	1.46E-08	3.01E-05
IL1B	2.25	11.748	19.06	4.53E-08	8.03E-05
FCAMR	4.45	3.6814	18.3	6.33E-08	8.16E-05
CCL3L1	2.89	6.0085	18.25	6.31E-08	8.16E-05
ANO8	2.71	7.1796	17.71	7.88E-08	9.24E-05
SRC	2.69	6.737	17.27	9.55E-08	9.24E-05
CCL5	2.2	8.5712	17.12	1.02E-07	9.24E-05
SOCS3	5.63	2.6873	16.28	1.58E-07	0.00013
IL23A	5.24	2.8044	16.03	1.76E-07	0.00013
SLC43A2	2.55	5.6425	15.98	1.71E-07	0.00013
RRAD	2.54	5.6183	15.01	2.74E-07	0.00018
MMP12	4.04	3.3013	14.6	3.44E-07	0.0002
CXCL8	2.44	9.8427	14.55	3.45E-07	0.0002
CXCL3	3.28	4.6141	14.55	3.49E-07	0.0002
DLL4	3.1	4.6097	14.04	4.53E-07	0.00023
IGFBP5	3.37	3.5561	13.93	4.82E-07	0.00023
CLEC5A	2.32	5.8277	13.44	6.26E-07	0.00027
CCL3	2.11	7.9598	13.13	7.43E-07	0.0003
TBX2	3.67	3.5867	13.12	7.55E-07	0.0003
IGSF6	3.13	3.6038	12.79	9.05E-07	0.00034
MGAT4C	4.68	2.7007	12.36	1.21E-06	0.00042
PLVAP	3.46	4.2472	12.27	1.24E-06	0.00042
TLR8	3.65	3.2149	12.14	1.35E-06	0.00044
LST1	1.92	7.0373	12.01	1.43E-06	0.00046
BCAR1	2.01	7.2131	11.8	1.63E-06	0.0005
PLAU	2.23	6.4015	11.75	1.69E-06	0.0005
CCL4	2.21	5.3422	11.74	1.69E-06	0.0005
CXCL2	3.94	2.8946	11.68	1.80E-06	0.00051
CDC42EP2	2.91	3.4647	11.46	2.04E-06	0.00056
RGS16	1.76	8.8896	11.29	2.26E-06	0.00059
S100A9	2.22	5.0796	11.02	2.70E-06	0.00066
NRG1	1.85	7.2189	10.97	2.79E-06	0.00067
ECE1	3.49	3.5996	10.85	3.05E-06	0.00072
FP565260.3	2.37	5.5301	10.75	3.23E-06	0.00075
BCL11A	3.57	2.8148	10.73	3.32E-06	0.00076
CCL3L3	2.89	4.1014	10.66	3.44E-06	0.00077
TFPI2	2.18	7.3403	10.56	3.69E-06	0.00081

Gene	logFC	AveExpr	t	P.Value	adj.P.Val
RGCC	-2.94	7.40173	-24.13	7.55E-09	2.77E-05
APOE	-2.66	8.23535	-22.27	1.39E-08	3.01E-05
ATP9A	-3.36	5.57759	-22.1	1.48E-08	3.01E-05
ATF3	-3.2	5.1938	-18.67	5.31E-08	8.16E-05
PITPNM1	-3.14	5.97267	-17.52	8.62E-08	9.24E-05
ITGA3	-2.76	6.60015	-17.07	1.04E-07	9.24E-05
NRGN	-2.21	9.06169	-15.25	2.43E-07	0.00017
CD84	-3.06	4.88836	-14.98	2.79E-07	0.00018
COL9A2	-2.4	7.16614	-14.37	3.79E-07	0.00021
ATP6V1C2	-4.51	3.14635	-14.01	4.74E-07	0.00023
SELENON	-2.08	7.87809	-13.97	4.68E-07	0.00023
ADRA2B	-5.07	2.50615	-13.81	5.36E-07	0.00025
TSC22D1	-2.29	7.54702	-13.58	5.78E-07	0.00026
CCDC88B	-2.96	4.85105	-13.25	6.95E-07	0.00029
COL4A1	-2.84	4.12875	-12.95	8.26E-07	0.00032
DLX3	-5.63	2.0064	-12.38	1.23E-06	0.00042
EGR2	-2.48	5.99973	-12.37	1.15E-06	0.00042
COL4A2	-2.6	5.57505	-11.78	1.66E-06	0.0005
SIAE	-1.96	6.66154	-11.65	1.80E-06	0.00051
ITPKB	-2.35	5.48545	-11.44	2.05E-06	0.00056
VSIG2	-2.88	3.6101	-11.33	2.21E-06	0.00059
APOC1	-2.15	5.86775	-11.26	2.31E-06	0.00059
GPC4	-1.97	7.18018	-11.25	2.31E-06	0.00059
SLC7A8	-2.11	7.1764	-11.16	2.45E-06	0.00061
LPL	-1.99	9.41021	-10.61	3.57E-06	0.00079
ATP1B1	-2.33	4.4206	-10.28	4.48E-06	0.00093
FAM135B	-3.11	2.90962	-10.24	4.64E-06	0.00094
CD36	-2.34	7.26452	-10.24	4.61E-06	0.00094
HLA-F	-5.95	1.67045	-10.23	5.00E-06	0.00097
CDK18	-3.15	2.87876	-10.22	4.70E-06	0.00094
KLHL4	-4.09	2.01203	-10.19	4.88E-06	0.00096
MMP7	-3.24	3.02242	-10.04	5.36E-06	0.001
CRABP2	-6.29	0.81109	-9.949	6.20E-06	0.00107
MYRF	-2.25	4.46978	-9.879	5.97E-06	0.00107
SUSD3	-3.67	2.68752	-9.775	6.54E-06	0.00112
SCD	-1.73	7.2992	-9.53	7.72E-06	0.00126
TRPM8	-2.86	4.678	-9.521	7.81E-06	0.00126
IL2RG	-4.92	1.20355	-9.505	8.25E-06	0.00129
NES	-2.19	4.86899	-9.504	7.88E-06	0.00126
CUX2	-3.56	2.51371	-9.344	9.02E-06	0.00137

TREM1	2.96	3.1002	10.35	4.29E-06	0.00092
S100B	4.18	1.757	10.33	4.42E-06	0.00093
TNFAIP6	3.31	2.5925	10.12	5.04E-06	0.00097
NOS2	2.88	3.2613	10.08	5.16E-06	0.00098
IL6	7.09	-2.184	10	6.12E-06	0.00107
AREG	2.44	5.9935	10	5.46E-06	0.00101
C1orf21	2.13	6.05	9.859	6.05E-06	0.00107
CCL4L2	2.66	3.6748	9.848	6.11E-06	0.00107
SLC7A11	2.34	5.4075	9.674	6.94E-06	0.00117
SERPINE1	1.99	5.9174	9.609	7.28E-06	0.00122
C1QTNF1	6.49	0.0604	9.588	8.15E-06	0.00129
TNFAIP2	2.05	6.5529	9.559	7.56E-06	0.00125
MYCL	1.96	6.595	9.303	9.18E-06	0.00137
ORM1	3.12	2.6001	9.285	9.37E-06	0.00139
EREG	3.28	3.509	9.261	9.57E-06	0.0014
UNC5B	2.07	5.9401	9.212	9.85E-06	0.00141
APCDD1	3.62	1.9566	9.187	1.02E-05	0.00145
GFPT2	3.11	2.4055	9.14	1.05E-05	0.00146
HSPA1A	2.05	5.1202	9.038	1.13E-05	0.00156
LAMP3	6.68	0.5757	9.008	1.28E-05	0.00167
IGSF8	1.95	6.3841	8.974	1.19E-05	0.00162
SH3BP5	1.91	5.612	8.913	1.25E-05	0.00167
CSF2	7.4	-1.244	8.896	1.44E-05	0.00182
EHD1	2.11	8.8284	8.883	1.28E-05	0.00167
OSM	2.88	2.5985	8.852	1.31E-05	0.0017
DMRT2	2.62	3.2257	8.752	1.42E-05	0.00182
MAS1	3.94	1.4062	8.723	1.48E-05	0.00183
PDZD4	3.45	2.7252	8.674	1.53E-05	0.00185
EGR1	1.85	6.1766	8.627	1.57E-05	0.00189
TRAF1	1.75	6.4884	8.585	1.62E-05	0.00192
DTX4	1.93	6.029	8.529	1.70E-05	0.002
C22orf42	2.57	3.333	8.464	1.80E-05	0.00208
STK26	1.96	5.3967	8.431	1.85E-05	0.0021
SIGLEC17P	2.02	4.9142	8.419	1.86E-05	0.0021
SH3PXD2B	1.7	6.4687	8.409	1.88E-05	0.0021
AC106865.1	2.87	2.4808	8.39	1.92E-05	0.00213
BMP8A	2.55	3.6027	8.292	2.08E-05	0.00229
PTGS2	3.14	3.2668	8.18	2.30E-05	0.00246
CLDN1	3.36	1.7224	8.18	2.31E-05	0.00246
OR2F1	6.53	0.5893	8.159	2.58E-05	0.00265
FJX1	2.28	4.4186	8.11	2.43E-05	0.00257
PID1	2.17	4.0145	8.095	2.46E-05	0.00258
ATP6V0D2	2.08	4.7736	8.085	2.48E-05	0.00258
F3	2.51	4.5356	8.067	2.52E-05	0.00261

ELK1	-2.7	3.35602	-9.342	8.94E-06	0.00137
HSD3B7	-3.12	2.83636	-9.323	9.11E-06	0.00137
CHODL	-3.55	2.31872	-9.253	9.67E-06	0.0014
FN1	-1.59	10.0277	-9.148	1.04E-05	0.00145
SLCO2B1	-1.99	6.52249	-8.947	1.21E-05	0.00164
SPRY2	-1.67	7.35353	-8.879	1.28E-05	0.00167
MYL1	-6.58	-2.29424	-8.841	1.46E-05	0.00182
RUBCNL	-2.65	4.12709	-8.72	1.46E-05	0.00182
LIPA	-1.74	8.17777	-8.684	1.50E-05	0.00183
FBLIM1	-2.74	3.52957	-8.604	1.61E-05	0.00191
NR2F1	-3.17	2.83549	-8.499	1.76E-05	0.00205
ESAM	-2.71	3.00602	-8.426	1.86E-05	0.0021
CORO2B	-2.63	3.51804	-8.276	2.11E-05	0.0023
KCNH4	-2.45	3.61062	-8.223	2.20E-05	0.00239
RHPN1	-2.02	4.5077	-7.992	2.69E-05	0.00274
ANKRD10	-1.98	4.87616	-7.948	2.79E-05	0.00281
PTGDS	-4.81	1.02654	-7.902	3.04E-05	0.00296
ZNF219	-2.05	4.34782	-7.865	3.00E-05	0.00294
GIPC3	-1.82	5.07167	-7.82	3.12E-05	0.003
CARNS1	-3.05	2.39695	-7.781	3.26E-05	0.00308
SEL1L3	-2.22	4.14779	-7.746	3.34E-05	0.00314
MAP3K7CL	-2.27	4.09945	-7.711	3.45E-05	0.00322
NPHP3-ACAD11	-5.8	-2.68178	-7.613	4.07E-05	0.00371
DOP1B	-1.63	6.4959	-7.607	3.78E-05	0.00351
BCAN	-3.41	2.60203	-7.593	3.88E-05	0.00355
LGI2	-1.85	5.33032	-7.579	3.88E-05	0.00355
CAPN11	-5.75	-2.70619	-7.422	4.85E-05	0.00425
CD300LB	-3.15	1.86064	-7.393	4.64E-05	0.00414
CACNA1A	-1.85	5.23764	-7.362	4.74E-05	0.0042
SPSB1	-1.72	5.59642	-7.229	5.37E-05	0.00462
GSG1L	-4.66	1.68789	-7.157	6.01E-05	0.00504
SORL1	-1.83	4.93877	-7.156	5.75E-05	0.00486
HCN2	-2.62	2.94209	-6.99	6.76E-05	0.00558
METTL7B	-2.49	2.99412	-6.972	6.88E-05	0.00564
CYP19A1	-1.9	5.93753	-6.919	7.23E-05	0.00589
SYP	-2.93	1.80167	-6.9	7.41E-05	0.00595
DDIT4L	-1.67	7.15228	-6.827	7.91E-05	0.00627
HMX3	-5.64	-1.97126	-6.788	8.89E-05	0.00671
MAN1C1	-1.89	5.03809	-6.776	8.32E-05	0.00643
FAM53B	-1.88	4.34049	-6.773	8.34E-05	0.00643
CAMK2B	-3	1.67984	-6.76	8.51E-05	0.00649
ISM1	-3.24	1.69836	-6.74	8.71E-05	0.00661
DOCK6	-1.98	4.68298	-6.641	9.52E-05	0.00707
CHI3L1	-1.36	11.8176	-6.572	0.000102	0.0075

SERPINB7	5.36	0.3441	7.994	2.86E-05	0.00284
IL1A	3.15	2.3636	7.965	2.77E-05	0.00281
CCDC30	1.95	4.411	7.923	2.85E-05	0.00284
GPR82	2.89	2.4741	7.875	2.99E-05	0.00294
VEGFC	1.79	5.4151	7.839	3.07E-05	0.00296
BHLHE40	1.63	6.3985	7.787	3.22E-05	0.00306
ADAM19	3.68	2.039	7.418	4.58E-05	0.00411
IRAK2	1.64	6.1113	7.41	4.53E-05	0.00409
NLRP2	2.29	3.2568	7.337	4.85E-05	0.00425
HBEGF	1.58	7.8641	7.314	4.95E-05	0.00431
SNAI1	2.11	3.6865	7.299	5.02E-05	0.00435
PLAT	5.3	-0.708	7.225	5.75E-05	0.00486
MYO10	1.71	6.8822	7.215	5.44E-05	0.00465
NCF1C	1.73	6.1076	7.083	6.17E-05	0.00515
ICOSLG	1.8	6.8253	6.991	6.74E-05	0.00558
MYO1B	1.75	6.3046	6.894	7.40E-05	0.00595
CACNA1E	2.41	6.3475	6.893	7.42E-05	0.00595
STC1	2.34	4.7799	6.859	7.68E-05	0.00612
SLC16A10	2.7	2.0189	6.819	8.00E-05	0.00628
LPXN	1.49	7.845	6.815	8.00E-05	0.00628
WNT5B	1.93	4.9603	6.806	8.08E-05	0.0063
PPFIA2	2.09	4.1249	6.756	8.48E-05	0.00649
CALML5	5.52	-2.969	6.73	9.38E-05	0.00701
GABRG1	3.43	0.8843	6.687	9.22E-05	0.00692
ADGRE3	5.24	-1.032	6.642	0.000102	0.0075
IER3	1.46	7.6376	6.506	0.000109	0.00794
S100A8	3.14	1.3973	6.479	0.000113	0.00816
HS3ST1	2.35	4.724	6.452	0.000116	0.00824
GPR141	3.33	0.9905	6.422	0.00012	0.00855
DSEL	3.79	0.6036	6.41	0.000123	0.00865
PLPP1	2.88	2.2634	6.32	0.000133	0.00918
NCF1	1.52	8.0807	6.301	0.000135	0.00918
HMOX1	1.44	7.8093	6.283	0.000138	0.00929
MCTP2	4	-0.077	6.238	0.000148	0.00987
BIRC3	1.92	4.0866	6.182	0.000153	0.01014
GTPBP3	1.77	5.6071	6.127	0.000162	0.01061
CRLF2	5.09	-3.184	6.121	0.000174	0.01111
SAMSN1	2.25	2.6846	6.092	0.000168	0.01088
SLC12A7	1.88	5.427	6.091	0.000169	0.01088
PMP22	3.25	0.7937	6.085	0.000172	0.01102
NCF1B	1.74	4.6078	6.041	0.000178	0.01126
GPR68	1.6	7.814	6.029	0.00018	0.01136
ISG20	2.18	3.5499	6.024	0.000181	0.01138
COL1A1	2.3	2.5262	6.018	0.000183	0.01141
KCNN3	3.53	0.1825	5.917	0.000207	0.01272

MAPK13	-1.96	4.2381	-6.542	0.000105	0.0077
PLCB2	-1.6	7.28941	-6.474	0.000113	0.00816
LRRC4B	-3.14	2.47049	-6.471	0.000114	0.00819
STAC2	-2.93	1.52976	-6.401	0.000122	0.00864
CXorf57	-4.58	1.67132	-6.389	0.000129	0.009
IGHD	-5.52	-1.98366	-6.38	0.000134	0.00918
ACTA1	-5.7	-0.45411	-6.356	0.000139	0.00932
MAGED1	-3.33	0.82168	-6.333	0.000132	0.00918
FABP4	-1.81	6.90621	-6.303	0.000135	0.00918
SLC37A1	-2	3.65256	-6.299	0.000135	0.00918
LSP1	-2.09	4.20574	-6.255	0.000142	0.00949
MAP4K2	-2.16	2.87614	-6.164	0.000156	0.0103
RB1	-1.56	6.05068	-6.145	0.000159	0.01045
OR7E47P	-5.17	-3.00189	-6.108	0.000177	0.01125
PTPRN2	-1.84	4.0833	-6.09	0.000169	0.01088
AC244260.1	-5.07	-3.04655	-6.051	0.000187	0.01167
PRSS36	-2.01	4.05973	-5.979	0.00019	0.01178
SDC3	-1.7	6.33596	-5.89	0.00021	0.01282
KANK2	-1.78	5.48133	-5.843	0.000221	0.01333
SHMT1	-2.63	1.6524	-5.82	0.000227	0.0136
BEX5	-4.88	-3.14179	-5.812	0.000242	0.01417
AL645935.2	-4.84	-3.16044	-5.808	0.000243	0.01417
ATP13A2	-1.6	5.53458	-5.785	0.000235	0.01401
TMPRSS9	-3.12	0.99853	-5.724	0.000254	0.01467
GBGT1	-1.99	3.20426	-5.718	0.000254	0.01467
PPARG	-1.69	5.13031	-5.701	0.000258	0.01483
LAMP1	-1.42	7.44504	-5.682	0.000264	0.01504
TLN2	-2.34	3.15496	-5.667	0.000269	0.01526
SLC49A3	-2.12	2.92392	-5.629	0.00028	0.01578
P2RX7	-3.33	0.86234	-5.604	0.000292	0.01627
AZU1	-1.72	5.88835	-5.593	0.000292	0.01627
MAF	-1.73	5.31392	-5.588	0.000294	0.01627
NUAK1	-2.94	1.2211	-5.576	0.0003	0.01654
PGAM2	-4.79	-3.18325	-5.551	0.000324	0.01754
SH2D3C	-2.34	3.60184	-5.537	0.000312	0.01701
KCNQ1	-2.03	4.95741	-5.525	0.000315	0.01715
MTCO2P25	-4.74	-3.20887	-5.511	0.000338	0.01818
PCDH1	-3.2	0.64595	-5.494	0.00033	0.01782
KRT17P2	-4.91	-2.28729	-5.464	0.000359	0.01903
GNG7	-2.24	2.31544	-5.445	0.000346	0.01852
F11R	-1.61	5.11692	-5.427	0.000353	0.01875
DYSF	-2.81	1.36307	-5.36	0.000384	0.02013
MYH1	-4.6	-3.28299	-5.355	0.000404	0.02089
WIPF3	-1.63	5.40791	-5.353	0.000385	0.02013
AL591806.3	-1.71	4.14731	-5.35	0.000386	0.02013

CXCL6	2.89	1.7452	5.916	0.000205	0.01265
EVA1A	2.69	1.6256	5.887	0.000211	0.01285
SDC1	1.72	4.8215	5.875	0.000213	0.01291
IGSF10	5.03	-2.664	5.839	0.000236	0.01401
TFAP2C	2.29	2.4868	5.837	0.000222	0.01337
SV2C	5.33	-0.742	5.827	0.000242	0.01417
ROBO1	1.56	6.4463	5.771	0.000239	0.01413
ERBB3	5.33	-1.17	5.754	0.000262	0.01501
COL5A3	1.91	3.6397	5.724	0.000252	0.01464
AP006222.1	2.72	1.5938	5.641	0.000278	0.0157
IGDCC4	2.78	1.1749	5.627	0.000282	0.01583
SOD2	1.36	9.2023	5.549	0.000307	0.01686
OTULINL	1.66	4.4762	5.539	0.00031	0.01699
MAP1LC3C	2.51	1.6438	5.435	0.00035	0.0187
AC140134.1	4.74	-3.358	5.403	0.000384	0.02013
CCL19	4.67	-3.396	5.354	0.000405	0.02089
DLL1	2.24	3.2249	5.238	0.00044	0.02228
NPFFR2	2.27	2.3065	5.178	0.000473	0.02358
MFSD2A	1.34	9.638	5.176	0.000474	0.02358
EVX1	4.27	-0.944	5.095	0.000544	0.02607
S100A3	2.25	1.9263	5.01	0.000579	0.02757
MEIKIN	2.58	1.1098	5.009	0.000581	0.02757
CD1A	1.77	3.3651	4.949	0.000623	0.02916
ORM2	2.83	0.719	4.945	0.000631	0.02943
EPHB3	2.34	2.6798	4.913	0.000653	0.03026
EFNA5	2.53	2.4814	4.893	0.000669	0.03084
PCDH18	2.72	2.2116	4.872	0.000688	0.03161
ROBO4	1.79	6.2465	4.852	0.000702	0.03215
MMP13	2.94	0.3214	4.844	0.000715	0.03256
ROR2	1.85	3.3261	4.769	0.000778	0.03461
AL592429.1	1.9	2.7883	4.765	0.000782	0.03468
TNFSF10	2.83	0.5784	4.737	0.000816	0.03596
CTTNBP2	3.06	0.5303	4.706	0.000851	0.03717
RIPOR2	2.85	0.6275	4.676	0.000881	0.03825
AC131206.1	2.12	2.1081	4.65	0.000904	0.03901
GPR157	2.35	3.0758	4.641	0.000917	0.03942
PRR32	2.17	2.0239	4.6	0.000964	0.04096
HS3ST3A1	1.46	6.3314	4.595	0.000969	0.04105
SIGLEC7	1.54	4.6585	4.552	0.001024	0.04252
FGF2	1.82	4.7574	4.55	0.001026	0.04252
TBX15	2.38	1.2293	4.524	0.001064	0.04365
CPXM1	1.99	2.1905	4.484	0.001118	0.04506
MARCKSL1	1.31	9.6327	4.471	0.001137	0.04569
C1orf61	4.37	-1.328	4.469	0.001199	0.047
EHF	3.01	0.6452	4.461	0.001165	0.04616

KALRN	-4.15	-0.35602	-5.331	0.000409	0.02094
FADS2	-1.67	4.92164	-5.331	0.000394	0.0205
CHIT1	-3.23	1.64592	-5.302	0.000414	0.02103
NR1H3	-1.57	5.21588	-5.301	0.000408	0.02094
COL11A2	-2.85	1.86061	-5.301	0.000412	0.021
AC091167.1	-4.7	-3.22897	-5.237	0.000466	0.02336
ZBTB7B	-1.6	7.12405	-5.236	0.000441	0.02228
PHLDA3	-1.76	4.5688	-5.21	0.000455	0.0229
FA2H	-4.29	-1.47431	-5.193	0.000484	0.02377
BBOX1	-4.09	0.0109	-5.175	0.000491	0.02395
PARP4	-1.61	4.74458	-5.164	0.00048	0.02377
ERFE	-1.84	3.59921	-5.158	0.000484	0.02377
TRNP1	-2.61	1.72401	-5.158	0.000486	0.02378
ITGA7	-1.83	3.86166	-5.158	0.000484	0.02377
POU3F1	-4.62	-3.26672	-5.15	0.000516	0.02489
ADCY7	-1.69	5.26439	-5.133	0.000499	0.02425
AC009690.3	-4.91	-3.11924	-5.128	0.000535	0.02573
ZNF385A	-1.38	6.94269	-5.109	0.000513	0.02484
PCDH11Y	-5.67	-1.07169	-5.078	0.000585	0.02769
TRIM54	-2.15	2.68799	-5.019	0.000572	0.02734
USP2	-1.52	6.15709	-4.993	0.00059	0.02783
CXCL16	-1.8	4.00093	-4.961	0.000614	0.02885
BACH2	-3.25	0.83614	-4.944	0.000636	0.02959
MYOZ1	-1.94	2.96726	-4.905	0.000657	0.03039
SETDB2	-1.72	3.78448	-4.836	0.000716	0.03256
PRAM1	-1.64	4.10672	-4.825	0.000726	0.03289
ENC1	-1.91	4.62183	-4.8	0.000749	0.03386
GDF15	-1.35	8.40371	-4.788	0.00076	0.03422
FAM84B	-2.05	2.46536	-4.785	0.000763	0.03424
SLC27A3	-1.71	3.98983	-4.783	0.000765	0.03424
GFI1	-2.14	3.16329	-4.775	0.000773	0.0345
NTNG2	-4.18	-1.87912	-4.767	0.000813	0.03593
WNK2	-3.05	0.40316	-4.723	0.000833	0.0366
NUP210	-1.51	6.84835	-4.702	0.000846	0.03707
GPC3	-2.01	2.80212	-4.687	0.000863	0.03755
KCNMA1	-2.38	1.92417	-4.66	0.000894	0.03869
IGF2	-4.21	-3.04584	-4.636	0.000961	0.04094
FCER2	-4.72	-0.41162	-4.635	0.00098	0.04138
TACC2	-1.54	4.95807	-4.628	0.000929	0.03985
ITM2B	-1.46	7.7675	-4.605	0.000957	0.0409
TNNC2	-3.98	-1.63801	-4.6	0.000998	0.04203
PATJ	-4.24	-3.04545	-4.598	0.00101	0.04228
ARHGEF37	-4.94	-0.81449	-4.594	0.001042	0.04285
DLX4	-3	0.17455	-4.565	0.001017	0.04245
HAVCR2	-1.38	7.17233	-4.563	0.001009	0.04228

KITLG	1.62	4.0182	4.454	0.001162	0.04616
GPR183	1.57	4.3717	4.444	0.001177	0.04654
ATP10A	1.93	5.9472	4.384	0.001273	0.04963

BIVM-ERCC5	-2.16	3.08472	-4.55	0.001028	0.04252
IRS2	-1.5	6.21863	-4.541	0.001039	0.04285
IGF2BP3	-4.64	-0.39451	-4.54	0.001104	0.0448
SHISA2	-4.29	-2.64019	-4.501	0.001147	0.0457
SLC29A1	-1.47	5.14751	-4.497	0.001099	0.0448
RAB20	-2.6	0.83776	-4.496	0.001105	0.0448
ARGLU1	-1.52	5.29572	-4.495	0.001101	0.0448
SPNS3	-2.31	1.7147	-4.485	0.001118	0.04506
DIRAS2	-1.97	2.48334	-4.468	0.001141	0.0457
TBC1D10C	-2.18	2.09796	-4.465	0.001146	0.0457
NOTCH3	-2.88	0.29765	-4.447	0.001182	0.04659
NOXA1	-2.23	1.73497	-4.438	0.001188	0.04671
LRP5	-1.85	3.77912	-4.408	0.001233	0.0482

S3 Table. KEGG pathways enriched by the genes downregulated in the *RORA*-knockout

ID	Pathway	N	Up	Down	P.Up	P.Down
hsa03320	PPAR signaling pathway	49	1	7	0.46	3E-06
hsa04974	Protein digestion and absorption	51	3	7	0.026	4E-06
hsa04512	ECM-receptor interaction	60	3	7	0.040	1E-05
hsa04979	Cholesterol metabolism	33	0	5	1	7E-05
hsa05200	Pathways in cancer	412	16	15	7.E-05	0.0004
hsa04725	Cholinergic synapse	79	0	6	1	0.0006
hsa04972	Pancreatic secretion	53	0	5	1	0.0007
hsa04971	Gastric acid secretion	54	1	5	0.498	0.0007
hsa04911	Insulin secretion	54	1	5	0.49	0.0007
hsa05165	Human papillomavirus infection	273	5	11	0.26	0.001
